# Modified amplatz sheath with suction versus standard sheath in percutaneous nephrolithotomy for treating large renal stones > 2 cm: a prospective randomized trial

**DOI:** 10.1007/s00345-025-05489-5

**Published:** 2025-04-25

**Authors:** Abdallah Fathi, Mohammed Ibrahem, Shabib Mohammed, Mostafa Mohamed, Kareem ElAttar

**Affiliations:** Urology Department, Faculty of Medicine, Benha University, Benha, 13511 Qalyubiya Governorate Egypt

**Keywords:** Percutaneous nephrolithotomy, Amplatz sheath, Large kidney stones, Stone-free rate, Operative time

## Abstract

**Purpose:**

To evaluate the efficacy and safety of using a modified amplatz as an access sheath in percutaneous nephrolithotomy (PCNL) compared to the standard Amplatz sheath for the treatment of renal stones larger than 2 cm.

**Methods:**

This prospective randomized study was conducted on 240 patients with renal stones larger than 2 cm who underwent PCNL. Patients were randomized into two equal groups: Group I (standard Amplatz sheath) and Group II (modified Amplatz sheath). Outcomes measured included operative time, stone-free rate (SFR), complications, and hospital stay.

**Results:**

The modified Amplatz sheath group had a significantly shorter mean operative time compared to the standard sheath group (56 ± 12 vs. 83 ± 17 min, respectively; *P* < 0.001). The SFR was higher in the modified sheath group (90.8% vs. 80%; *P* = 0.017). Postoperative complications, such as fever (4.2% vs. 10.8%) and pain (13.3% vs. 20.8%), were significantly lower in the modified sheath group (*P* = 0.036). The modified sheath group also had a shorter hospital stay, with 93.3% discharged within two days compared to 85% in the standard group (*P* = 0.038). Multivariate analysis indicated that the use of the modified sheath reduced the risk of residual stones by 91% (OR = 0.086, 95% CI = 0.027–0.280, *P* < 0.001).

**Conclusions:**

The use of a modified Amplatz sheath in PCNL significantly reduces operative time, increases the SFR, and decreases postoperative complications compared to the standard Amplatz sheath.

## Introduction

Percutaneous nephrolithotomy (PCNL) has become the gold standard for the management of large renal stones, particularly those exceeding 2 cm, due to its high efficacy and relatively low morbidity [1, 2].

Traditionally, PCNL involves the use of an Amplatz sheath to access the renal collecting system, allowing for stone fragmentation and removal [3]. Despite its effectiveness, the traditional approach carries certain risks, such as extended operative times, significant bleeding, and postoperative complications, including infections and prolonged hospital stays [4, 5].

Numerous attempts have been made to enhance the PCNL technique to overcome these disadvantages. While these efforts have led to various improvements, they have not fully resolved all the challenges associated with the procedure [6–8].

In this study, we compared the use of a modified amplatz sheath as an access sheath with the standard Amplatz sheath in PCNL to evaluate whether this novel technique represents a significant improvement in treating renal stones larger than 2 cm. Our objective was to assess the efficacy and safety of modified amplatz sheath in terms of operative time, stone-free rates, and postoperative complications.

## Patients and methods

### Design and population

This prospective, randomized clinical trial was carried out on 240 patients with renal stones larger than 2 cm who underwent PCNL at the Urology Department of Benha University Hospital between May 2023 and May 2024.

### Patient selection

The study included patients aged 18–70 years with renal stones larger than 2 cm. Patients were excluded if they were children, had stones smaller than 2 cm, were pregnant, had abnormal kidney anatomy such as pelvic kidney, or had other pathologies such as untreated urinary tract infections (UTIs) or potential renal tumors.

### Randomization and treatment

Patients were randomized using a computer-generated randomization table into two equal groups: **Group I**: Patients underwent PCNL using the standard Amplatz sheath (30 Fr). **Group II**: Patients underwent PCNL using a modified amplatz sheath.

### Preoperative assessment

All patients underwent thorough preoperative evaluations, including:

Complete history taking, general examination, local examination, laboratory investigations, abdominopelvic ultrasound (US), computed tomography of the urinary tract (CT-UT), and plain abdominal radiograph (KUB).

### Operative technique

All procedures were performed under general anesthesia. Initially, a retrograde ureteral catheter (6 Fr) was inserted via a diagnostic cystoscope to the renal pelvis or upper calyx in the dorsal lithotomy position and fixed to a urethral catheter. Patients were then positioned prone for the PCNL procedure.

**Group I (standard Amplatz Sheath)**: The kidney was accessed through a retrograde study with dye injection to identify the targeted calyx. An 18-gauge metal needle was used for calyx puncture under C-arm fluoroscopic guidance. A guidewire was inserted, followed by fascial dilation up to 30 Fr, and the standard Amplatz sheath (30 Fr) was used as the access sheath. **Group II (modified amplatz sheath)**: The same initial steps were followed as in Group I, but the modified Amplatz sheath was used as an access sheath over the 30 Fr fascial dilator. The standard Amplatz sheath was modified by incorporating a screw mechanism to allow the addition of a 10 mm laparoscopic port component.

### This modification consists of two channels

**Main Channel**: This channel is used for introducing the 26 Fr nephroscope and is equipped with a side valve to prevent fluid leakage around the nephroscope. The valve allows manual control of irrigation to maintain visibility during the procedure. **Side Channel**: This channel is utilized for suction, enabling precise control of irrigation fluid and the removal of small stone fragments. This feature ensures a clear surgical field, reduces intra-renal pressure, and facilitates efficient stone clearance.

**Fig. 1:**
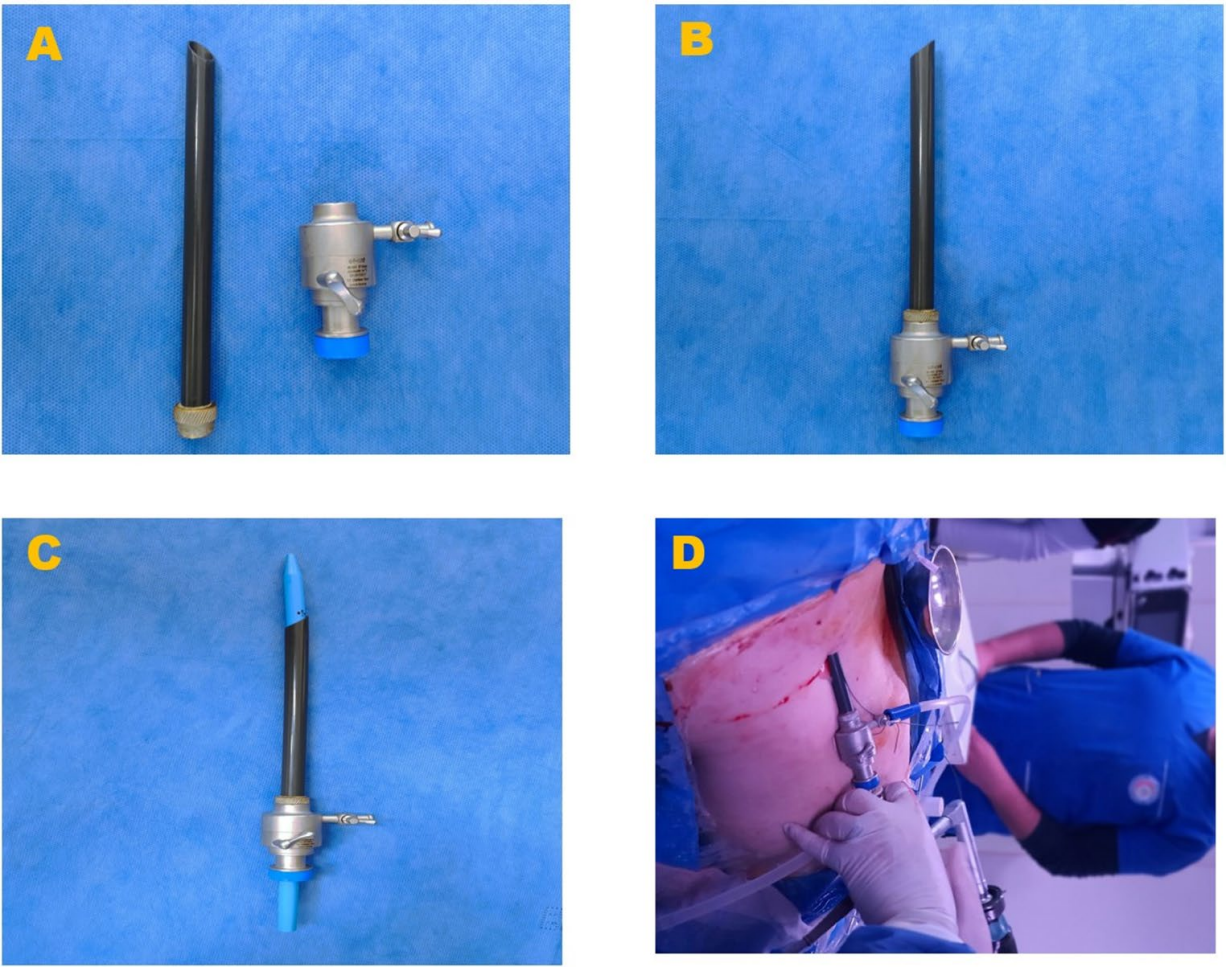
Multi panel graph showing modified amblatz sheath and its use in PCNL, (**A**) Modified Amplatz Sheath Components as standard sheath with adding part from laparoscopic port which contain manual valve for introduce nephroscope 26 fr. and side channel for control irrigation and using suction, (**B**) Assembled Modified Amplatz Sheath, (**C**) Modified Amplatz Sheath with dilator 30 fr., and (**D**) Using Suction During PCNL

A 26 Fr nephroscope with a 30 Fr operating sheath was employed for stone fragmentation using a pneumatic lithoclast and stone extraction with forceps. Adequate irrigation flow was maintained throughout the procedure to ensure visibility and surgical precision. Postoperatively, a 24 Fr nephrostomy tube was placed for drainage and removed after 2–3 days. Urethral catheters were removed 24 h post-nephrostomy tube removal if no urine leakage was observed. Figure 1 illustrates the components of the modified Amplatz sheath and its use during PCNL.

### Outcome measures

The primary outcome measures were SFR, operative time, postoperative complications, including fever, infection, and bleeding, and length of hospital stay.

### Sample size estimation

The sample size was calculated using G*power software version 3.1.9.2 based on a previous study by **Tuoheti et al.**, who reported a stone free rate of 85.3% and 70.3% in D-mPCNL and C-mPCNL groups, respectively [8]. The total sample size needed to detect such a difference was 240 patients (120 per group). Alpha and power were adjusted at 0.05 and 0.8, respectively.

### Statistical methods

Data management and statistical analysis were done using SPSS version 28 (IBM, Armonk, New York, United States). Quantitative data were assessed for normality using the Kolmogorov-Smirnov test and direct data visualization methods. According to normality, quantitative data were summarized as means and standard deviations. Categorical data were summarized as numbers and percentages. Quantitative data were compared between the studied groups using the independent t-test. Categorical data were compared using the Chi-square or Fisher’s exact test. Univariate and Multivariate logistic regression analyses were done for treatment to predict stone-free rate. The odds ratios with 95% confidence intervals were calculated. All statistical tests were two-sided. P values less than 0.05 were considered significant.

## Results

**Fig. 2:**
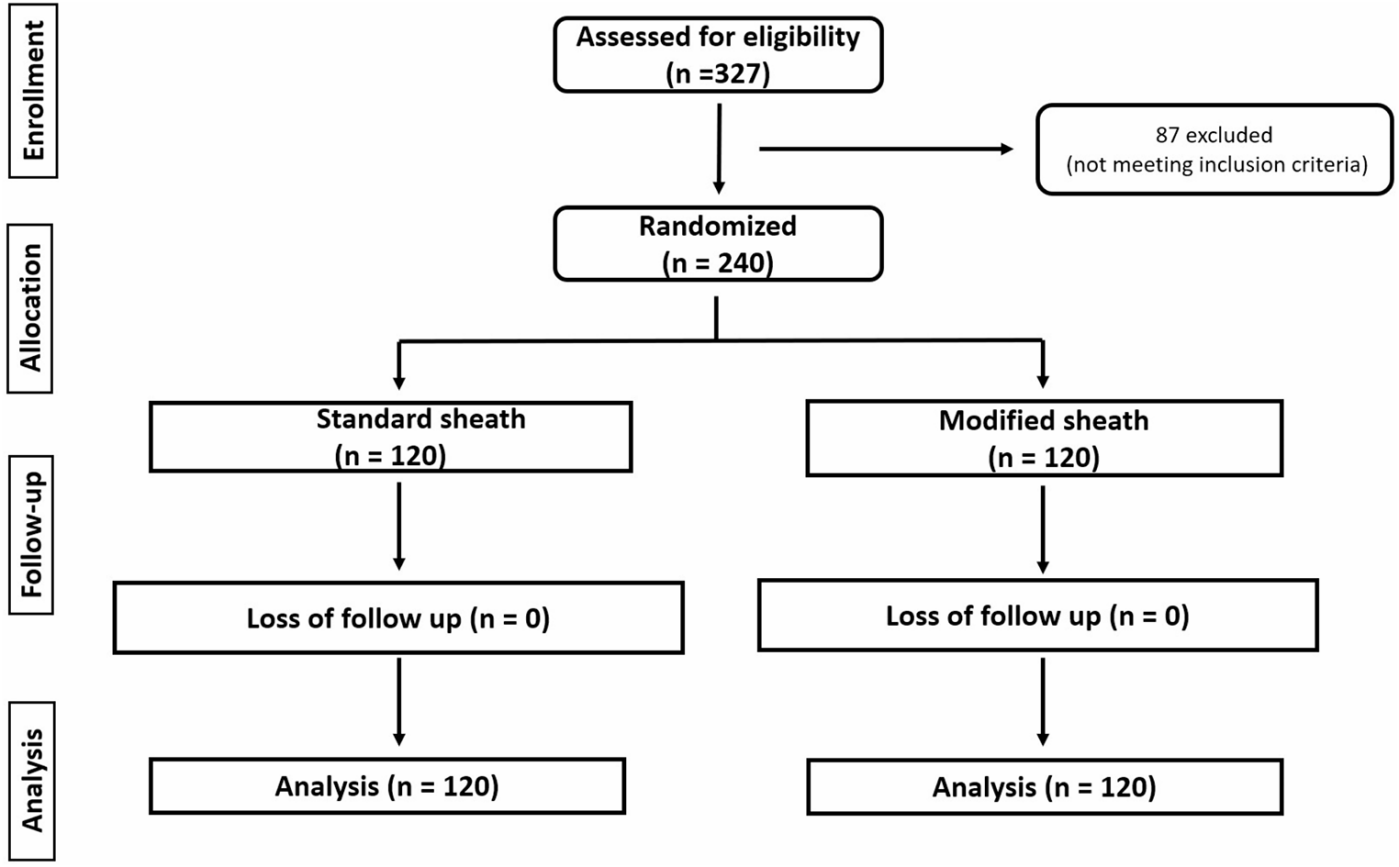
CONSORT flow diagram of the studied patients

In this trial, 327 patients were evaluated for eligibility; 87 did not meet the inclusion criteria. The remaining 240 patients were randomly assigned into two groups of equal size, with 120 patients in each group. All patients were then followed up, and no loss of follow-up was reported. The data from all patients were included in the final statistical analysis. Figure 2.

General characteristics and operative and postoperative findings are shown in Tables 1 and 2.

**Table 1:** General and baseline characteristics of the studied groups

		Group I (*n* = 120)	Group II (*n* = 120)	*P*-value
**Age (years)**	Mean ± SD	40 ± 7	39 ± 7	0.773
**Gender**				
Males	n (%)	59 (49.2)	49 (40.8)	0.194
Females	n (%)	61 (50.8)	71 (59.2)	
**BMI**	Mean ± SD	24.1 ± 0.6	24.1 ± 0.7	0.819
**Stone site**				
Lower calyx	n (%)	55 (45.8)	58 (48.3)	0.580
Pelvis	n (%)	55 (45.8)	56 (46.7)	
Lower calyx and pelvis	n (%)	10 (8.3)	6 (5)	
**Stone side**				
Right	n (%)	55 (45.8)	60 (50)	0.518
Left	n (%)	65 (54.2)	60 (50)	
**Stone size (cm)**	Mean ± SD	2.7 ± 0.3	2.6 ± 0.3	0.337
**Stone density**	Mean ± SD	889 ± 166	884 ± 167	0.807

SD: Standard deviation; BMI: Body Mass Index; cm: centimeters

**Table 2:** Operative and postoperative findings in the studied groups

		Group I (*n* = 120)	Group II (*n* = 120)	*P*-value
**Operative time (min)**	Mean ± SD	83 ± 17	56 ± 12	< 0.001*
**Blood transfusion**	n (%)	6 (5)	4 (3.3)	0.518
**Stone free rate**	n (%)	96 (80)	109 (90.8)	0.017*
**Residual size (cm)**	Mean ± SD	0.7 ± 0.2	0.7 ± 0.2	0.374
**Complications**				
Fever	n (%)	13 (10.8)	5 (4.2)	0.036*
Bleeding required bl. Transfusion	n (%)	7 (5.8)	3 (2.5)	
Pain	n (%)	25 (20.8)	16 (13.3)	
UTI	n (%)	7 (5.8)	6 (5)	
No complications	n (%)	68 (56.7)	90 (75)	
**Clavien-Dindo classification**				
Grade I	n (%)	24 (46.2)	16 (53.3)	0.531
Grade II	n (%)	28 (53.8)	14 (46.7)	
**Hospital Stay (days)**				
Two days	n (%)	102 (85)	112 (93.3)	0.038*
Three days	n (%)	18 (15)	8 (6.7)	
**Auxiliary procedure †**				
ESWL	n (%)	17 (14.8)	9 (7.6)	0.127
URS	n (%)	1 (0.9)	1 (0.8)	
2nd look	n (%)	1 (0.9)	0 (0)	
No need	n (%)	96 (83.5)	109 (91.6)	
**Final stone-free rate†**	n (%)	115 (100)	119 (100)	-

*Significant P-value; † Five patients were lost to follow-up in group I and one patient in group II; SD: Standard deviation; min: minutes; cm: centimeters; n (%): number (percentage); UTI: Urinary Tract Infection; ESWL: Extracorporeal Shock Wave Lithotripsy; URS: Ureteroscopy

### Prediction of residual stones

**Fig. 3:**
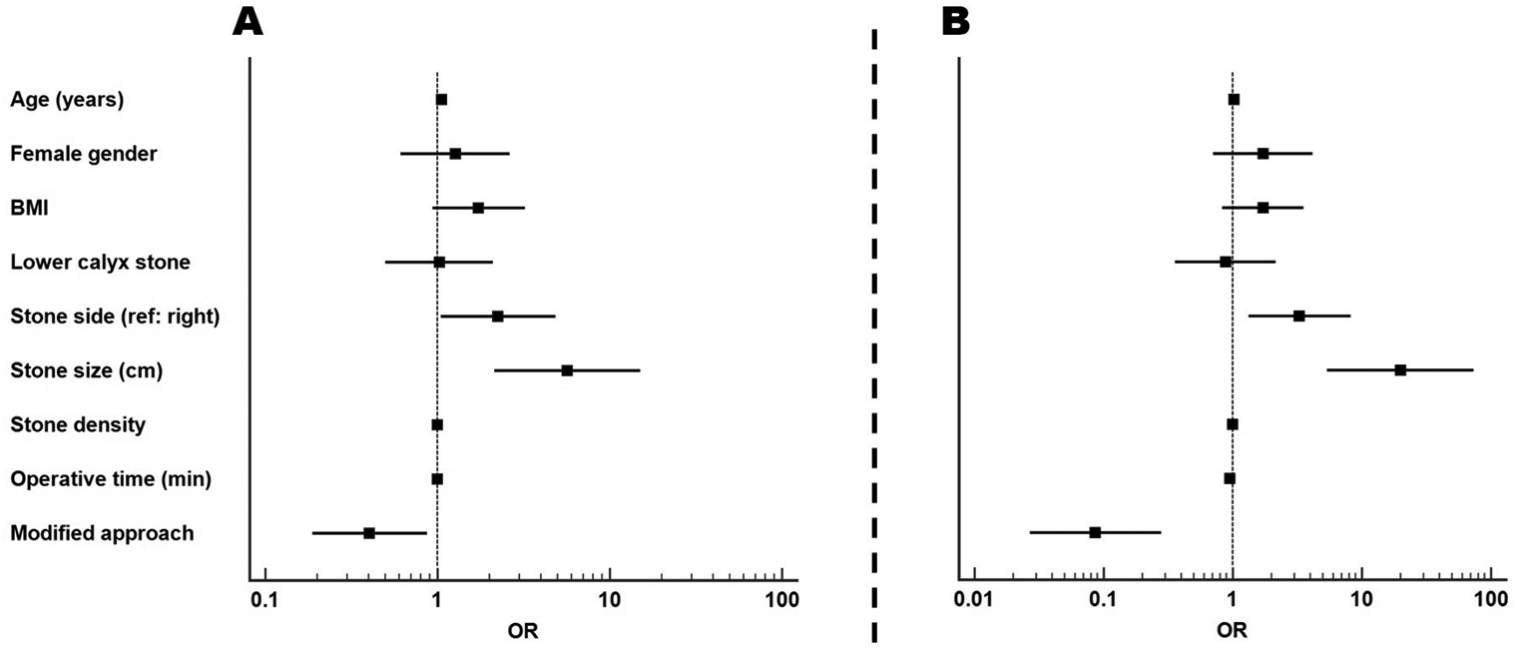
Univariate and multivariate analysis for prediction of residual stones

Univariate and multivariate logistic regression analysis was done to predict the stone-free rate. All general characteristics were included as predictors. On the univariate level, age, stone side, stone size, and using modified sheath were significant predictors of stone-free rate. On multivariate analysis, left-sided stone was associated with three times increased risk of residual stone (OR = 3.29, 95% CI = 1.324–8.176, *P* = 0.01), increased stone size by 1 cm was associated with 20 times increased risk of residual stone (OR = 19.934, 95% CI = 5.406–73.506, *P* < 0.001), prolonged operative time was associated with 5% reduced risk of residual stone (OR = 0.949, 95% CI = 0.921–0.978, *P* < 0.001), and using modified sheath was associated with about 91% risk reduction of residual stone (OR = 0.086, 95% CI = 0.027–0.280, *P* < 0.001). Table 3; Fig. 3.

**Table 3:** Univariate and multivariate analysis to predict the stone-free rate

	Univariate					Multivariate	
	OR (95% CI)	*P*-value			OR (95% CI)		*P*-value
**Age (years)**	1.062 (1.008–1.12)	**0.025**			1.028 (0.966–1.094)		0.381
**Female gender**	1.27 (0.612–2.636)	0.521			1.719 (0.708–4.174)		0.232
**BMI**	1.735 (0.938–3.21)	0.079			1.716 (0.833–3.535)		0.143
**Lower calyx stone**	1.026 (0.499–2.106)	0.945			0.88 (0.36–2.151)		0.780
**Stone side (ref: right)**	2.247 (1.046–4.825)	**0.038**			3.29 (1.324–8.176)		**0.01**
**Stone size (cm)**	5.675 (2.136–15.075)	**< 0.001**			19.934 (5.406–73.506)		**< 0.001**
**Stone density**	1.001 (0.999–1.003)	0.179			1.001 (0.999–1.004)		0.26
**Operative time (min)**	1.001 (0.983–1.019)	0.953			0.949 (0.921–0.978)		**< 0.001**
**Modified approach**	0.404 (0.188–0.867)	**0.02**			0.086 (0.027–0.28)		**< 0.001**

*Significant P-value; OR: Odds Ratio; CI: Confidence Interval; BMI: Body Mass Index; cm: centimeters; min: minutes

## Discussion

To our knowledge, this study represents the first direct comparison of standard Amplatz and modified Amplatz sheaths with suction in PCNL for large renal stones. Our findings demonstrate that the modified Amplatz sheath significantly enhances procedural efficiency and patient outcomes compared to the standard sheath. Specifically, the modified sheath group exhibited a shorter operative time, higher SFR, and fewer postoperative complications, including reduced rates of fever and pain. These improvements can be attributed to the design of the modified sheath, which allows for controlled irrigation, improved visualization, reduced intra-renal pressure, and efficient stone fragment clearance through suction.

Suction-assisted techniques in PCNL have been explored previously, highlighting their potential benefits. In agreement with our findings, **Tuoheti et al.** [8] evaluated the efficacy of a novel double-sheath negative-pressure PCNL (D-mPCNL) compared to conventional PCNL (C-mPCNL) for large kidney stones. Their findings showed that the D-mPCNL group had a significantly shorter operative time (41.97 ± 8.24 min vs. 52.30 ± 13.72 min, *P* < 0.001), a higher primary SFR (85.3% vs. 70.3%, *P* = 0.038), and lower rates of postoperative fever (2.9% vs. 14.1%, *P* = 0.021).

Interestingly, a meta-analysis by **Wang et al.** indicated that laparoscopic pyelolithotomy (LPL) and PCNL are both effective and safe for large renal pelvic calculi, with LPL showing higher SFRs and lower incidences of bleeding and postoperative fever compared to PCNL. Their study also noted shorter operative times and hospital stays in the PCNL group, but fewer hemoglobin drops in the LPL group [9]. **Eshghi et al.** reported the successful implementation of laparoscopy-assisted PCNL for treating pelvic kidney stones, highlighting the potential of laparoscopic techniques in managing complex renal stones [10]. Similarly, Santos et al. demonstrated the effectiveness of videolaparoscopy-guided percutaneous transperitoneal nephrolithotripsy, further supporting the benefits of enhanced visualization and access provided by laparoscopic methods [11].

Recently, in their systematic review, **Nizzardo et al.** [12] examined suction-assisted nephrostomic sheaths in PCNL. They observed SFRs ranging from 71.3 to 100% and complication rates from 1.5 to 38.9%. They also reported a trend towards better SFRs and lower complication rates in suction techniques. Furthermore, they noted a consistent 19-minute reduction in operative time with suction procedures.

Parallel to our results, **Zhu et al.** [13] compared the treatment outcomes of suctioning MPCNL and traditional MPCNL for renal staghorn stones. They conducted a matched-pair analysis and found that the suctioning MPCNL group achieved a significantly higher SFR after a single procedure (78.5% vs. 69.1%) and had a significantly shorter operative time (106.2 ± 18.4 vs. 132.1 ± 22.2 min) compared to the traditional MPCNL group. Additionally, the suctioning MPCNL group experienced fewer overall complications (16.8% vs. 27.3%) and a lower incidence of fever.

While suction techniques are not novel, our study introduces a practical and cost-effective modification to the standard Amplatz sheath. This modification integrates a laparoscopic port component, enabling simultaneous suction and controlled irrigation without requiring specialized equipment, making it an accessible advancement for clinical practice.

The use of a 30 Fr sheath and 24 Fr nephrostomy in all cases was chosen to accommodate the large stone burden (> 2 cm), facilitating effective stone fragmentation and clearance. Although miniaturized PCNL approaches have gained popularity for smaller stones, the standard 30 Fr sheath remains the contemporary standard of care for large renal stones due to its ability to handle larger fragments and reduce the need for auxiliary procedures.

The modified Amplatz sheath provides several theoretical advantages that contribute to its efficacy. By enabling precise control of irrigation fluid, it ensures a consistently clear surgical field, which enhances visualization during the procedure. Additionally, the suction mechanism reduces intra-renal pressure, thereby minimizing the risk of complications such as pyelovenous backflow and systemic absorption of irrigation fluid. These features collectively contribute to shorter operative times by improving visibility and facilitating the efficient removal of stone fragments [14, 15].

In practice, the benefits observed in this study align with these theoretical advantages. Patients in the modified sheath group demonstrated significantly higher stone-free rates and a reduced need for auxiliary procedures compared to those in the standard sheath group. Operative times were shorter, and postoperative complications, such as fever and infection, were notably decreased. Furthermore, the use of the modified sheath resulted in shorter hospital stays, with the majority of patients being discharged within two days, highlighting its practical utility in improving patient outcome.

Despite the promising findings, this study has several limitations. Firstly, the study was conducted at a single center with a relatively small sample size. Additionally, the study duration may not have allowed for the long-term assessment of stone recurrence rates and the durability of outcomes. Thus, future multi-center studies with larger sample sizes and standardized protocols may enhance the validity and the reproducibility of the results.

## Conclusions

Using modified amplatz sheath as an access sheath in PCNL significantly improves stone-free rates, reduces operative times, and minimizes complications, making it a superior alternative to traditional methods for managing large renal stones.

## Data Availability

No datasets were generated or analysed during the current study.
